# Fluorescence Bar-Coding and Flowmetry Based on Dark State Transitions in Fluorescence Emitters

**DOI:** 10.1021/acs.jpcb.3c06905

**Published:** 2023-12-21

**Authors:** Elin Sandberg, Baris Demirbay, Abhilash Kulkarni, Haichun Liu, Joachim Piguet, Jerker Widengren

**Affiliations:** Royal Institute of Technology (KTH), Experimental Biomolecular Physics, Dept. Applied Physics, Albanova University Center, 106 91 Stockholm, Sweden

## Abstract

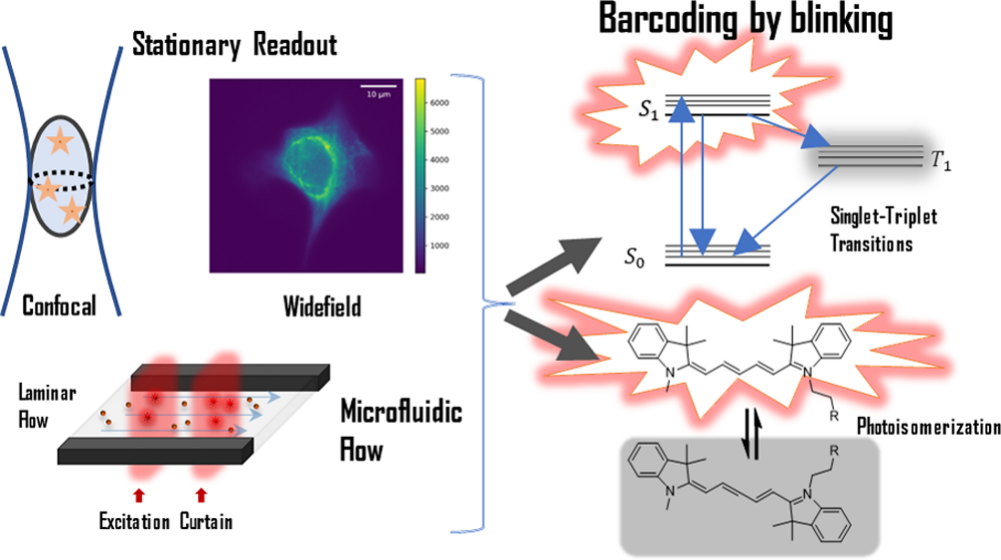

Reversible dark state transitions in fluorophores represent a limiting factor in fluorescence-based ultrasensitive spectroscopy, are a necessary basis for fluorescence-based super-resolution imaging, but may also offer additional, largely orthogonal fluorescence-based readout parameters. In this work, we analyzed the blinking kinetics of Cyanine5 (Cy5) as a bar-coding feature distinguishing Cy5 from rhodamine fluorophores having largely overlapping emission spectra. First, fluorescence correlation spectroscopy (FCS) solution measurements on mixtures of free fluorophores and fluorophore-labeled small unilamellar vesicles (SUVs) showed that Cy5 could be readily distinguished from the rhodamines by its reversible, largely excitation-driven *trans–cis* isomerization. This was next confirmed by transient state (TRAST) spectroscopy measurements, determining the fluorophore dark state kinetics in a more robust manner, from how the time-averaged fluorescence intensity varies upon modulation of the applied excitation light. TRAST was then combined with wide-field imaging of live cells, whereby Cy5 and rhodamine fluorophores could be distinguished on a whole cell level as well as in spatially resolved, multiplexed images of the cells. Finally, we established a microfluidic TRAST concept and showed how different mixtures of free Cy5 and rhodamine fluorophores and corresponding fluorophore-labeled SUVs could be distinguished on-the-fly when passing through a microfluidic channel. In contrast to FCS, TRAST does not rely on single-molecule detection conditions or a high time resolution and is thus broadly applicable to different biological samples. Therefore, we expect that the bar-coding concept presented in this work can offer an additional useful strategy for fluorescence-based multiplexing that can be implemented on a broad range of both stationary and moving samples.

## Introduction

In fluorescence readouts for drug screening and cellular and molecular studies in general, multiplexing strategies are used to increase the number of different cells or molecules that can be simultaneously identified and analyzed in a sample. A common barcoding strategy is to use fluorophores with different emission bands.^1,2^ However, spectral overlap in the emission bands and constraints in filters, dichroic mirrors, and excitation light sources in the instruments set a limit for how many color channels can be used in parallel. Even with the best fluorescence microscopes available, it is difficult to include more than six color channels.^2^ To extend the multiplexing capacity beyond colors, additional encoding dimensions have thus been employed, where use of different intensity levels in the spectral channels^1,2^ and dye localization within the encoded entities (e.g., nanoparticles or nucleic acid constructs)^3,4^ represent major strategies. They however typically require careful and elaborate design of carrier structures, whose sizes also make them difficult to use more generally as labels in biological samples.^1^ Other strategies include encoding by differences in fluorescence lifetimes,^5^ fluorescence anisotropies (FA),^6^ binding/unbinding kinetics,^7^ and Förster resonance energy transfer (FRET).^8^ Such multiplexing strategies can be well applied in specialized setups for e.g. single molecule spectroscopy^9^ and super-resolution imaging^7^ but come with instrumental demands, on light sources and detectors as well as on data acquisition/processing, which limit their more general applicability. Photochemical encoding, where the relative amounts of fluorophores emitting in different emission bands can be regulated by light activation, offers a multiplexing strategy with lower instrumentation demands but can only be applied on a few, specifically designed photoactivatable dyes^10,11^ which to date are not generally available. Overall, it is motivated to consider additional encoding strategies, which can complement and offer selective advantages over existing ones.

In this work, we show how reversible, photoinduced, dark state transitions, found in almost all fluorophores, can be used as an additional encoding dimension for fluorescence barcoding and multiplexing. These transitions have attracted attention in several different fields of fluorescence-based research. In fluorescence-based single molecule spectroscopy, population of dark transient states, such as triplet, photoisomerized, and photoionized states, constitutes a major limiting factor. In this context, fluorescence correlation spectroscopy (FCS) offers a straightforward method to analyze these transitions in fluorophores, to optimize excitation and sample conditions.^12−14^ In fluorescence super-resolution imaging on the other hand, essentially all techniques have the switching of fluorophores into nonemissive states as a basis for their operation.^15^ As a third aspect, the typically long lifetimes of transient dark states, such as triplet and photoisomerized states, as compared to the fluorescence lifetimes of the same fluorophores, make these states highly environmentally sensitive. Monitoring the blinking such states generate, by single-molecule, FCS, or transient state (TRAST) spectroscopy,^16^ can thus form the basis for microenvironmental sensing applications. More recent examples include FCS-monitored quantum dot (QD) blinking to probe QD dimerization following mRNA hybridization^17^ single-molecule blinking measurements of QDs and organic fluorophores as a basis for multiplexing^18^ or of different fluorophore-labeled DNA molecules, designed to have differences in their electron transfer rates.^19^ By transient state (TRAST) monitoring, such states can be followed by a relatively simple approach, from how the time-averaged fluorescence intensity from the fluorophores varies with the modulation of the laser excitation intensity.^16,20−22^ Since TRAST, in contrast to FCS, does not rely on single-molecule detection conditions or a high time resolution, it can be applied on a broader range of samples. Taking advantage of the environmental sensitivity of photoinduced triplet and redox states of fluorophores, TRAST measurements have been used to follow local changes in oxygen concentrations,^23^ pH,^24^ redox conditions,^25^ and low frequency molecular interactions,^26^ in cells and solutions.

Next to regular fluorescence parameters (intensity, F; emission wavelength, λ_em_; fluorescence lifetime, τ_f_; and fluorescence anisotropy, FA), we exploit in this work transitions of photoinduced, long-lived, dark states of different organic fluorophores as additional fluorescence-based identifiers. We show how fluorophores with close to identical emission spectra can be separately identified, based on different dark state transition properties, offering an additional encoding dimension for fluorescence bar-coding and multiple “colors” in fluorescence microscopy studies with limited spectral detection channels available. For demonstration, we first performed FCS measurements on CF640R, a rhodamine-based fluorophore, and Cy5, a pentamethine carbocyanine fluorophore, with very similar emission spectra. Using the same spectral detection channel and based on the characteristic photoisomerization of Cy5, a transition not found in CF640R, we show how the presence of CF640R can be accurately distinguished from Cy5, when the two fluorophores are mixed in solution or when labeled to lipid vesicles. We then performed TRAST experiments and showed that it is possible to distinguish Cy5 and CF640R on the same samples as well as when imaged in live cells, without requirements of high time resolution or single-molecule detection conditions. Finally, we established a microfluidic-based TRAST readout and demonstrated how dark transient states of fluorescence emitters can be characterized in an easy manner in flowing samples, and how the two fluorophores can be distinguished in flowing samples based on this information. This shows that transient state barcoding/multiplexing can be applied in a similar way for microfluidic measurements and also more generally indicates how additional transient state parameters can be added to flow-based readouts of molecules and cells, in addition to regular fluorescence parameters.

## Materials and Methods

### Preparation of Fluorophore Solutions and Lipid Vesicles

Fluorophore solutions and small unilamellar vesicles (SUVs) were prepared with Cy5 and CF640R, see Supplementary, Section S1 for details.

### Preparation of HEK293 Cells and Immunostaining

HEK293 kidney cells were prepared and immune-stained with Cy5 and Abberior Star 635 (AS635) as fluorophore labels; see Supplementary, Section S2 for details.

### Stationary Wide-Field TRAST Measurements

#### Theoretical Background

In TRAST measurements, fluorophore blinking kinetics are determined by recording the average fluorescence intensity, ⟨*F*⟩, from an ensemble of fluorophores subject to modulated excitation. With the excitation modulation systematically varied on the time scales of the fluorophore dark-state kinetics, rapid blinking kinetics can be quantified without the need for time-resolved detection.^20,21^ This enables wide-field cellular imaging of μs blinking kinetics using a regular camera and exposure times of seconds. For a fluorophore subject to a rectangular excitation pulse starting at *t* = 0, the fluorescence signal recorded in our experimental setup can be described by
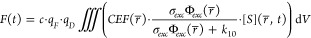
1Here, [*S*] denotes the probability that the fluorophore is in an excitable/emissive singlet state (either its ground, S_0_, or excited, S_1_, singlet state), *q*_*D*_ denotes the overall detection quantum yield of the emission from S_1_, *q*_*F*_ is the fluorescence quantum yield, and *k*_10_ is the the overall decay rate from S_1_. *CEF*(*r̅*) is the collection efficiency function of the detection system, and *c* is the fluorophore concentration.

At the onset of excitation, *F*(*t*) will show a characteristic relaxation on a μs to ms time scale, reflecting changes in the population of [*S*] (see eq 1), following transitions into dark transient (triplet, photoisomerized, or photoionized) states. Similar relaxations can also be observed in the time-averaged fluorescence signal resulting from a rectangular excitation pulse of duration *w*
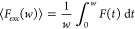
2when *w* is increased from the μs to the ms time range. Analyzing how ⟨*F*_*exc*_(*w*)⟩ varies with *w* then allows the population kinetics of long-lived photoinduced states of the fluorophore to be determined, which is the general basis for TRAST monitoring.^16,20−22^ To obtain sufficient photon counts, even for short *w*, we collected the total signal resulting from an excitation pulse train of *N* identical pulse repetitions. *N* is adjusted to maintain a constant laser illumination time, *t*_*ill*_ = *N*·*w*, typically 1–10 ms, for all *w*. A so-called TRAST curve is then produced by calculating ⟨*F*_*exc*_(*w*)⟩ for each pulse train, normalized for a given pulse duration, *w*_0_
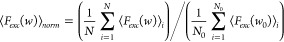
3For the normalization, *w*_0_ is chosen to be short enough (typically sub-μs) not to lead to any noticeable buildup of dark transient states, yet longer than the antibunching rise time of *F*(*t*) upon onset of excitation, which typically is in the nanosecond time range.^27^ In the above expression, ⟨*F*_*exc*_(*w*)⟩_*i*_ represents the total signal collected from the *i*th pulse in the pulse train, as defined in eq 2. A low excitation duty cycle, here η = 0.01, was used to allow the fluorophores to largely recover to S_0_ before the onset of the next pulse. The summations in eq 3 are then no longer required, and the expression simplifies further. By the normalization step of eq 3, several parameters cancel out, so that the final expression for ⟨*F*_*exc*_(*w*)⟩_*norm*_ is independent of *c*, *q*_*D*_, and *q*_*F*_.

#### Experimental Setup, Data Acquisition, and Analysis of Data

Wide-field TRAST measurements were carried out on a home-built TRAST setup, as previously described,^24,26^ with an inverted epi-fluorescence microscope (Olympus, IX70) and a 638 nm diode laser (Cobolt, 06-MLD) for excitation. The TRAST data was analyzed by Matlab software, as previously described.^26,28^ Parameter fitting was performed by simulating theoretical TRAST curves using eqs 1–3 and comparing them to the experimental data. The set of parameters best describing the experimental data was then found using nonlinear least-squares optimization. See Supplementary, Section S3 for further details.

### Wide-Field TRAST Imaging of Cells

For HEK293 cells stained with Cy5 and Abberior Star 635 dyes, images of TRAST amplitudes (*A*_*TRAST*_) and relaxation times (τ) were obtained by first acquiring pixel-wise TRAST curves. These TRAST curves were obtained from average fluorescence intensity values, ⟨*F*_*exc*_(*w*)⟩, generated upon pulse train excitations with different *w*, according to eq 2. In total, ⟨*F*_*exc*_(*w*)⟩ values were recorded for 30 different pulse trains with pulse widths, *w*, ranging between 100 ns (*w*_*short*_) and 1 ms (*w*_*ms*_).The recorded ⟨*F*_*exc*_(*w*)⟩ values were corrected for static ambient background and photobleaching and then normalized according to eq 3. In order to improve photon statistics and minimize the effects of stray photons, images were filtered with a 3 × 3 pixel Gaussian filter. The resulting pixel-wise TRAST curves (i.e., the sets of ⟨*F*_*exc*_(*w*)⟩_*norm*_ values) were then fitted using eqs 1–3, with the excitable/emissive singlet state, [*S*](t), in eq 1 fitted as a monoexponential function with relaxation amplitude *A*_*TRAST*_, relaxation time τ, and with [*S*](0) normalized to 1. Excitation rates in the sample were calculated as described in Supplementary section S4.

### Microfluidic TRAST Measurements

#### Experimental Setup

**Figure 1:**
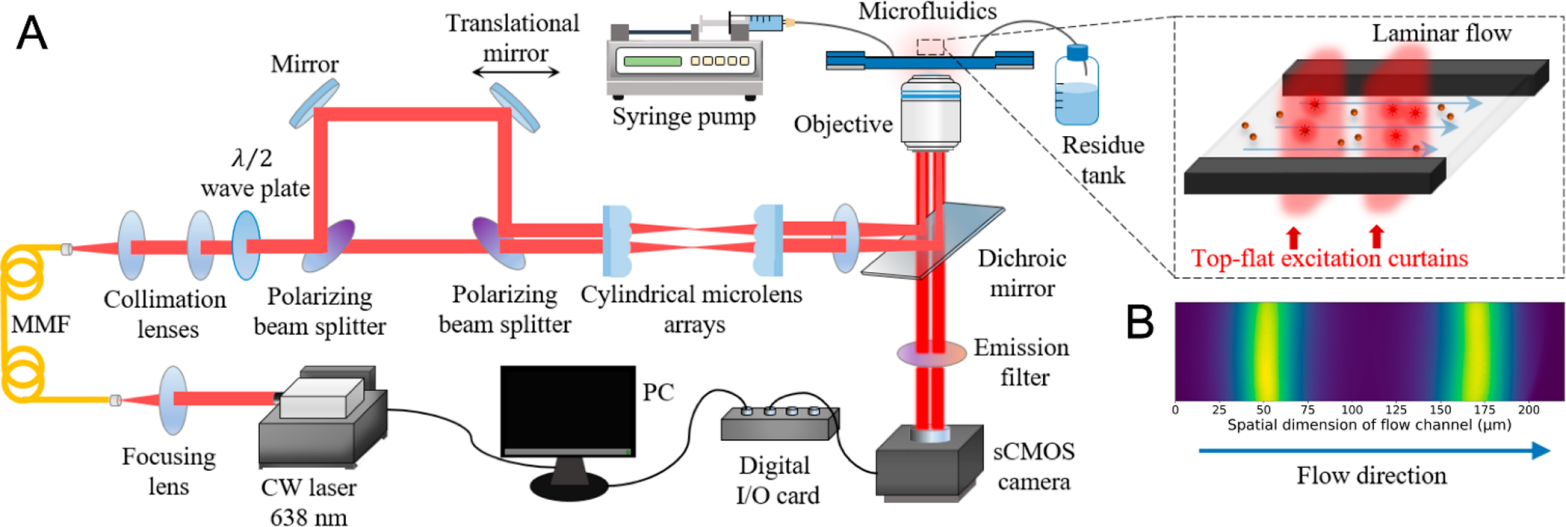
(A) A schematic representation of the microfluidic TRAST setup, see Materials and Methods for details. (B) Image of a CF640R solution in the microfluidic channel, illustrating the excitation intensity distribution on the flowed samples.

Experiments were established on a home-built wide-field TRAST setup (Figure 1A), with an inverted epi-fluorescence microscope (Olympus, IX73) and a 638 nm free-space, single longitudinal mode laser with a cleanup filter (0638L-41A-NI-NT-CF, Integrated Optics, Max. power ∼500 mW) for excitation. The laser beam was first focused into a 150 μm × 150 μm square-core multimode fiber, MMF (NA = 0.39, L101L02, Thorlabs), collimated using pairs of optical lenses, and then divided using a 50:50 polarizing beam splitter, PBS (PBSW-633, Thorlabs) arrangement to generate two parallel flat-top beams with equal excitation irradiances and beam dimensions. By a pair of reflective mirrors, the position of the second mirror could be translated to tune the distance between the excitation beams (Figure 1B). The two parallel beams were then fed through a pair of cylindrical microlens arrays (86-843, Edmund Optics) to control the spatial dimension and further improve their uniformity. The beams were then focused by a convex lens, reflected by a dichroic mirror (FF506-Di03, Semrock), and focused close to the back aperture of the microscope objective (Olympus, UPLSAPO 60x/1.20 W) to generate a wide-field illumination with two excitation “curtains” across the microfluidic channel containing the sample. Fluorescence was collected by the same microscope objective, passed through the same dichroic mirror and an emission filter (ET670/50m, Chroma) before detection by an sCMOS camera (Hamamatsu ORCA-Flash4.0 v2), triggered by a digital I/O card (PCI-6602, National Instruments).

In the microfluidic part of the setup, a mechanical syringe pump (KDS-200-CE, kdScientific) and a disposable medical syringe (5100-000 V0, HENKE-JECT) were used to control the flow through a 500 μm × 50 μm flow cell chip (FLC50, Micronit), with a thin bottom of borosilicate glass (D230) with reduced autofluorescence above 600 nm. The microfluidic chip was installed into a top-connect chip holder (purchased from Micronit) to ensure robust sealing and prevent chip cracking. Before each measurement session, the microfluidic system was flushed with methanol and ultrapure water (repeated 3 times) for cleaning.

#### Microfluidic TRAST Data Analysis

Fluorescence images of flowing samples passed over the two stationary excitation curtains were recorded with an sCMOS camera (Figure 1B). Each image was recorded for no more than 50 ms to prevent camera saturation, and a 4 × 4 pixel binning was applied by the camera software (Hokawo) to allow faster readout speeds and improved signal-to-noise ratios (S/N). The recorded fluorescence images were averaged every 600 frames, and ambient background was subtracted (from a blank solvent sample), yielding a mean fluorescence intensity image ⟨*F*(*x*, *y*)⟩, where *x* denotes the spatial coordinate along the flow channel, and *y* is the perpendicular coordinate, across the flow channel (Figure 1A). The ⟨*F*(*x*, *y*)⟩ images were then averaged across the mid part of the flow channel (30–86 μm), along *y*, where the flow rate was uniform and unilamellar. Thereby, we obtained an averaged fluorescence profile, ⟨*F̅*(*x*)⟩, with two peaks, representing the profile of the fluorescence generated after passage of fluorescent species through the two excitation beam curtains. With knowledge of the (laminar) flow rate, υ, determined from the set volume to be pumped per time unit by the mechanical syringe pump, divided by the cross-section area of the flow cell, and verified by FCS measurements, (Supplementary S5), ⟨*F̅*(*x*)⟩ can also be expressed with a time-dependence: ⟨*F̅*(*t*)⟩, where *t* = *x*/υ.

To determine the (stationary) excitation intensity field, Φ_*exc*_(*x*, *y*), in the flow-TRAST experiments, ⟨*F*(*x*, *y*)⟩ was determined for CF640R, at non-saturating excitation conditions (Φ_*exc*_ < 0.02 kW/cm^2^). The integral of Φ_*exc*_(*x*, *y*) over the two excitation beam curtains (each with a 15 μm 1/e-radius along *x* and 100 μm in length along *y*) was then calibrated to match the total power of the laser beam(s) after passage through the objective into the sample. Averaging along *y* then yielded the excitation intensity profile along the flow channel, Φ_*exc*_(*x*), which with *t* = *x*/υ then can also be expressed with a time dependence, Φ_*exc*_(*t*).

**Figure 2:**
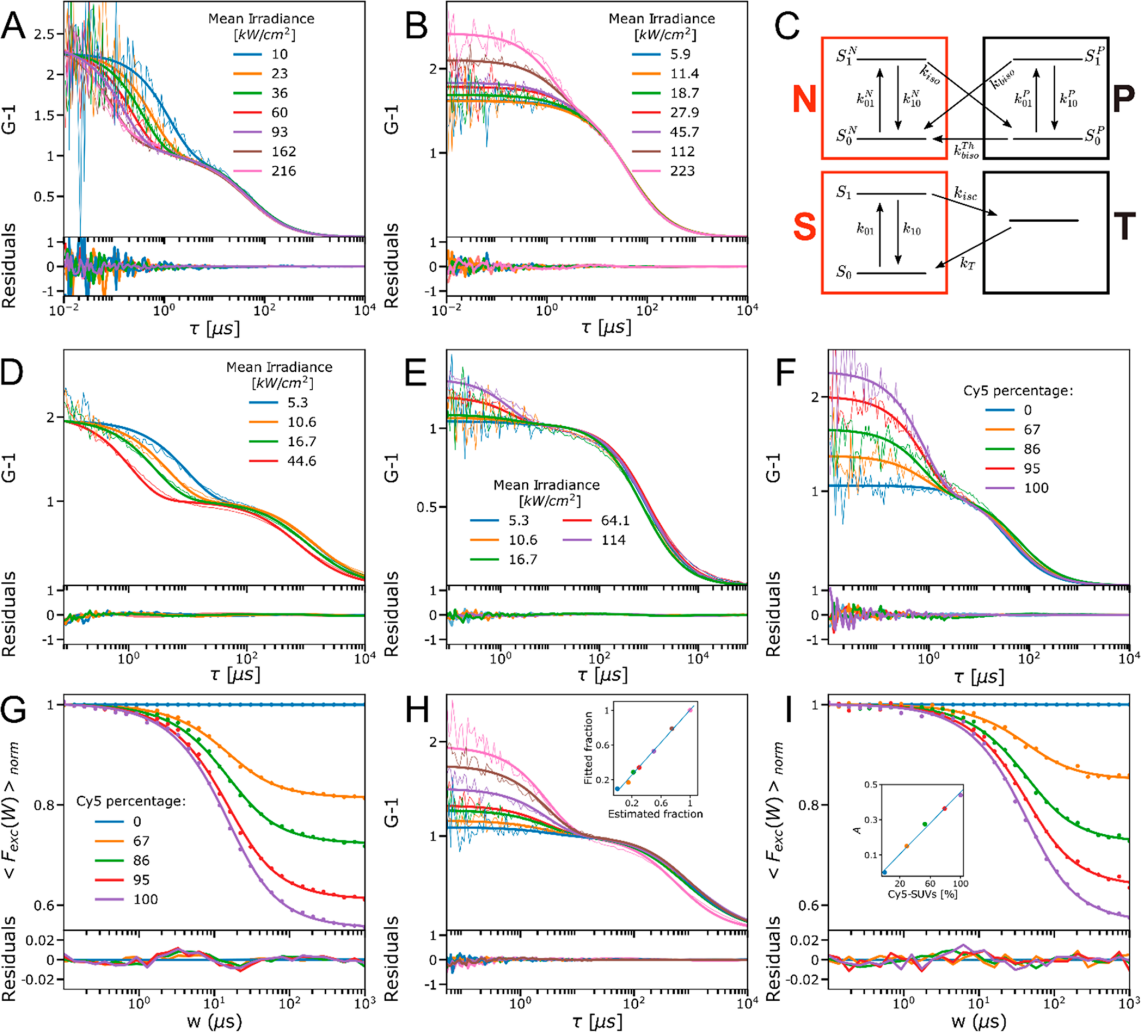
FCS data presented by thin lines recorded for free (A) Cy5 and (B) CF640R recorded at different mean Φ_*exc*_. Fitted curves are presented by thick solid lines with residuals given below. (C) Electronic state models for Cy5 (top) and CF640R (bottom). FCS measurements performed for SUVs labeled with (D) Cy5 and (E) CF640R at different mean Φ_*exc*_. (F) Experimental FCS curves (thin lines) and corresponding fitted curves (thick solid lines) from CF640R and Cy5 free dye mixtures. The percentages of Cy5 in the mixtures are indicated in the legend. Here, the mean Φ_*exc*_ was kept at 16 kW/cm^2^. (G) Experimental TRAST curves (dots) recorded from the same free CF640R and Cy5 dye mixtures as in Figure 2F, with the curves fitted to eq 14 (solid lines), using the Cy5 percentages determined by FCS (Figure 2F) and with the molecular brightness ratio *Q* = *Q*_*CF*_/*Q*_*Cy*5_ as a globally fitted parameter. Mean Φ_*exc*_: 1 kW/cm^2^. Fitting residuals are given below. (H) Experimental FCS curves (thin lines) and corresponding fitted curves (thick solid lines) from CF640R-SUVs and Cy5-SUVs mixtures. The inset shows how the estimated fractions of labeled Cy5-SUVs compare to the fitted ones. Mean Φ_*exc*_: 16 kW/cm^2^. (I) Experimental TRAST curves (dotted lines) and fitted curves (solid lines) from CF640R-SUVs and Cy5-SUVs mixtures. Mean Φ_*exc*_: 1 kW/cm^2^.

Based on Φ_*exc*_(*t*), we can then predict ⟨*F̅*(*t*)⟩ for different fluorophores and where the shape of ⟨*F̅*(*t*)⟩ will depend on the photodynamics and dark state buildup in the fluorophores as they pass through the excitation field(s) in the fluidic channel. With a peak Φ_*exc*_ of 3.4 kW/cm^2^ in the flow-TRAST experiments, excited state saturation is negligible (σ_*exc*_Φ_*exc*_ ≪ *k*_10_) for Cy5 and CF640R. For the same reason and given the low quantum yields of triplet state formation for these fluorophores, triplet state formation can also be neglected. Moreover, given the low Φ_*exc*_ applied, the limited passage times of the fluorophores through the excitation curtains, and since the Cy5 and CF640R dyes only flow through these curtains once, photobleaching can be neglected in the analyses. For CF640R, ⟨*F̅*(*t*)⟩ is then directly proportional to Φ_*exc*_(*t*):
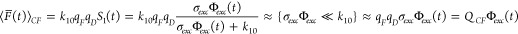
4Here, *Q*_*CF*_ = *q*_*F*_*q*_*D*_σ_*exc*_ denotes the detected brightness of CF640R. For Cy5, its photodynamics can be described by a two-state photoisomerization model, including a fluorescent *all*-*trans* state, *N*, and a dark photoisomerized *cis* state, *P* (Figure 2C, top) with effective rates of isomerization and back-isomerization between *N* and *P* given by^14^
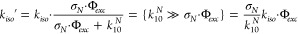
5
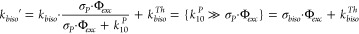
6Here, σ_*N*_ and σ_*P*_ denote the excitation cross sections of the singlet ground state of *N* and *P*, respectively, *k*_10_^*N*^ and *k*_10_^*P*^ denote their corresponding excited singlet state decay rates, and *k*_*iso*_ and *k*_*biso*_ signify the isomerization and back-isomerization rates. *k*_*biso*_^*Th*^ represents the thermal, not excitation-driven back-isomerization rate from *P* to *N*. Since *k*_*biso*_, *k*_10_^*P*^, and σ_*P*_ could not be individually determined, the back-isomerization from *P* to *N* can be defined as a cross section:
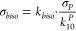
7From the model, the population buildup of *P* upon passage through the excitation curtains will influence ⟨*F̅*(*t*)⟩ as
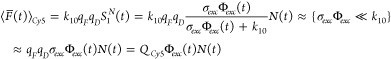
8where *N*(*t*) denotes the fraction of Cy5 fluorophores in an *all*-*trans* form, *N*, and *Q*_*Cy*5_ = *q*_*F*_*q*_*D*_σ_*exc*_ is the brightness of *N*. How *N*(*t*) evolves upon transit through the two excitation curtains can be described by
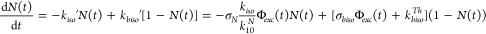
9and can be calculated recursively with the initial condition (when entering the first excitation curtain) *N*(0) = 1 and by knowledge of Φ_*exc*_(*t*).

In the analyses of the flow-TRAST data, we normalized Φ_*exc*_(*t*), ⟨*F̅*(*t*)⟩_*CF*_, and ⟨*F̅*(*t*)⟩_*Cy*5_ to unity at their peak values when passing the first excitation curtain in the flow channel, denoted as Φ̂_*exc*_(*t*), , and . Thereby, Φ̂_*exc*_(*t*) = , while for Cy5, the buildup of P upon passing the excitation fields in the flow channel can alter both the shape of  as well as the relative amplitude of its second emission peak, compared to the first one. For a mixture of CF640R and Cy5, we can then determine the relative fractions of CF640R and Cy5 passing through the flow channel by
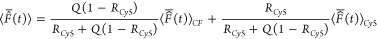
10where *R*_*Cy*5_ and (1 – *R*_*Cy*5_) are the fractions of Cy5 and CF640R fluorophores, and *Q* = *Q*_*CF*_/*Q*_*Cy*5_ is the relative fluorescence brightness of CF640R compared to that of Cy5.

Microfluidic TRAST data were analyzed by a home-built software implemented in Python. In the analyses, ⟨*F̅*(*t*)⟩ recorded from the samples with Cy5 were fitted to eq 8, with *N*(*t*) recursively calculated from eq 9. The set of rate parameters best matching the measurement data was obtained by Levenberg–Marquardt nonlinear least-squares optimization. In the data fitting, the singlet excited state lifetime of Cy5 was fixed to 1 ns. To determine the fraction of fluorophores in mixed samples, the recorded ⟨*F̅*(*t*)⟩ was fitted to eq 10 in the same manner, with *Q* determined beforehand,  determined as described above,  given by Φ̂_*exc*_(*t*), and with *R*_*Cy*5_ and the thermal relaxation rate (*k*_*biso*_^*Th*^) as the only fitted parameters.

### FCS Measurements and Analysis

FCS measurements were performed on a commercial, epi-illuminated, confocal laser scanning microscope (Olympus FV1200), with the samples excited by the focused beam of a 640 nm diode laser (LDH-D-C-640, PicoQuant GmbH, Berlin) and the emitted fluorescence collected through the same microscope objective (UPlanSApo 60*x*/1.2w, Olympus). The normalized autocorrelation of the recorded fluorescence intensity fluctuations (the FCS curves) typically displayed relaxation terms attributed to diffusion (*G*(τ)) and fluorophore relaxation into a dark transient state (*G*_T_(τ)) and were analyzed as previously described,^13,14^ using a Levenberg-Marquart nonlinear least-squares optimization, with no weighting on the residuals. See Supplementary, Section S6 for further details.

## Results and Discussion

### FCS and Stationary Wide-Field TRAST Measurements

FCS measurements were first performed on free Cy5 and CF640R in an aqueous solution (Figures 2A and 2B). From eqs S2–S4 and assuming uniform excitation intensities within the confocal detection volume, the recorded autocorrelation curves (FCS curves) for freely diffusing fluorophores undergoing reversible transitions into a dark state (a *cis* photoisomer or a triplet state) can be expressed as^13,14,29^
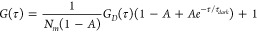
11where *N*_*m*_ is the average number of fluorescent molecules in the detection volume, *A* denotes the dark state amplitude (the steady-state fraction of fluorophores in the detection volume being in a reversible dark state), and τ_*dark*_ denotes the relaxation time of the dark state transitions. For Cy5 and CF640R, *A* and τ_*dark*_ showed quite different features and dependences on Φ_*exc*_.

For Cy5, a prominent, almost Φ_*exc*_ independent dark state relaxation amplitude was observed in the recorded FCS curves (Figure 2A), consistent with a two-state isomerization model with reversible excitation-driven transitions, between a fluorescent *trans* state, *N*, and a nonfluorescent *cis* photoisomer, P.^14^ The FCS curves, recorded at different Φ_*exc*_, were globally fitted to eq 11, based on the model in Figure 2C (top), including eqs 5–7. At the range of Φ_*exc*_ applied in the FCS experiments, . *k*_*biso*_^*Th*^ can then be set to zero in the fits. To account for a minor triplet state buildup observed for Φ_*exc*_ > 100 kW/cm^2^, the Cy5 model also included a triplet state, with *k*_*isc*_ fixed to 1.1 μs^–1^ and *k*_*T*_ fixed to 0.5 μs^–1^, according to previously found values.^14^ In the fitting, σ_*N*_ was set to 6.2 · 10^–16^ cm^2^,^14^ τ_*D*_ and *N*_*m*_ were allowed to vary freely, and *k*_*iso*_ and σ_*biso*_ were then fitted globally to 29 μs^–1^ and 0.15 × 10^–16^ cm^2^, respectively. The model and fitted *k*_*iso*_ and σ_*biso*_ values are well in agreement with previous data^14^ and could well reproduce the recorded FCS curves (Figure 2A).

For CF640R, lower dark state relaxation amplitudes were observed in the recorded FCS curves (Figure 2B) than for Cy5, which increased with higher Φ_*exc*_ and almost vanished at lower Φ_*exc*_ (<10 kW/cm^2^). This Φ_*exc*_ dependence is consistent with reversible transitions into a dark, triplet state, T, with a non-excitation dependent triplet decay rate, *k*_*T*_, and effective rate of intersystem crossing, *k*_*isc*_′, given by
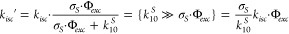
12Here, σ_*S*_ denotes the excitation cross section of the ground singlet state, S_0_, *k*_*isc*_ is the intersystem crossing rate from the excited singlet state, S_1_, and *k*_10_^*S*^ denotes the S_1_-to-S_0_ deexcitation rate. Using eq 12, the FCS curves from CF640R can then be described by a similar 2-state model to that of Cy5 (Figure 2C, bottom), including the states S (comprising S_0_ and S_1_) and T, with  excitation-driven but not *k*_*T*_. When fitting the CF640R data to eq 11, τ_*D*_ and *N*_*m*_ were again allowed to vary freely, σ_*S*_ was set to 4 · 10^–16^ cm^2^, while *k*_*isc*_ and *k*_*T*_ were globally fitted to 0.7 μs^–1^ and 0.5 μs^–1^, respectively. From the determined *N*_*m*_ and the fluorescence intensity registered in the FCS measurement, the molecular brightness of CF640R, *Q*_*CF*_, was found to be on average 2.2 times higher than that of Cy5, *Q*_*Cy*5_, which can be expected since around 50% of the fluorophores are in the dark cis-state at steady-state. Overall, the FCS data show quite different dark state amplitudes and relaxation times for the two fluorophores, with a prominent, almost Φ_*exc*_-independent dark state amplitude present in all FCS curves of Cy5 (attributed to *trans*-*cis* photoisomerization), not found in the corresponding curves from CF640R.

Then, SUVs labeled with single Cy5 or CF640R were measured under different Φ_*exc*_, and with the resulting FCS-data analyzed in the same way as for the free dye samples (Figures 2D and 2E). In these measurements, before the FCS curves from the detected fluorescence intensity time-traces were calculated, a threshold was applied to filter out bursts/spikes in these time-traces (see SI, Section S6 for details). While CF640R-SUVs yielded similar fitted *k*_*isc*_ and *k*_*T*_ rates as for free CF640R, the fitted isomerization rates determined for Cy5-labeled SUVs were lower than for free Cy5 (*k*_*iso*_ = 6.2 μs^–1^ and σ_*biso*_ = 0.042 × 10^–16^ cm^2^) but showed similar dark-state amplitudes, *A*. *A* did not increase with lower fractions of Cy5-labeled lipids in the SUV, indicating that the probability that any of the SUVs contained more than one fluorophore was negligible.^30^ The fitted rate parameter values from the FCS measurements of Cy5-SUVs and CF640R-SUVs, as well as for free Cy5 and CF640R, are summarized in Supplementary section S7. For CF640R-SUVs and Cy5-SUVs, *Q*_*CF*–*SUV*_ and *Q*_*Cy*5–*SUV*_ were found to be the same. This is due to a higher molecular brightness of Cy5 in the SUV samples, which can be explained by larger constraints and decreased isomerization rates for Cy5 in the SUVs. Since *k*_*iso*_ competes with the fluorescence decay-rate, a lower *k*_*iso*_ (and a correspondingly lowered transition rate to a twisted intermediate state between N and P) then results in a higher fluorescence quantum yield of Cy5.

Next, we performed FCS measurements of the free CF640R and Cy5 fluorophore mixtures (Figure 2F). In the recorded FCS curves, each fluorophore (*i*) will then contribute by its molecular brightness, *Q*_*i*_, squared
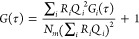
13where *R*_*i*_ is the fraction of the fluorophore *i*. The FCS curves were fitted with previously fitted parameters for Cy5 and CF640R fixed (from Figures 2A and 2B), and with τ_*D*_, *N*_*m*_, and *R*_*i*_ as freely fitted parameters. The fitted curves could well reproduce the data, with significant differences in the dark-state amplitudes and their dependence on *R*_*i*_ (Figure 2F).

We then measured the same free dye mixtures as recorded by FCS (Figure 2F) using TRAST (Figure 2G). With the much lower Φ_*exc*_ used in the TRAST experiments (1 kW/cm^2^, compared to 16 kW/cm^2^ in the FCS experiments), no triplet state buildup could be observed for CF640R. In contrast, for Cy5 (with both transitions to and from P being mainly excitation-driven), a prominent dark state amplitude was still observed. For a mixture of Cy5 and CF640R, and following eq 1, the detected fluorescence intensity at onset of a constant excitation intensity at time *t* = 0 is then proportional to
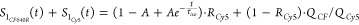
14where *S*_1_*CF*640*R*__ and *S*_1_*Cy*5__ represent the population probabilities of the excited singlet states of CF640R and Cy5, and *R*_*Cy*5_ is the fraction of Cy5 fluorophores. Integration of eq 14 over the pulse width, *w*, of the rectangular excitation pulse trains applied in the TRAST experiments, followed by normalization, then represents the data points of the TRAST curves, ⟨*F*_*exc*_(*w*)⟩_*norm*_ (eq 3). This was used to generate the best fit to the recorded TRAST curves in Figure 2G. In this fitting, *A* and τ_*iso*_ were first fitted for the TRAST curve recorded from the sample with 100% Cy5, which yielded *A* = 0.45 and τ_*iso*_ = 12.4 μs. Then *A* and τ_*iso*_ were fixed, and the molecular brightness ratio *Q*_*CF*_/*Q*_*Cy*5_ was globally fitted for all other curves, with *R*_*Cy5*_ allowed to vary 3% for each curve from the values determined for the mixed solutions from the FCS measurements (allowing for slight variations of the mixed solutions between the FCS and TRAST measurements). The fitted curves could well reproduce the experimental curves in Figure 2G, with *Q*_*CF*_/*Q*_*Cy*5_ fitted to 2.7, compared to 2.2 as obtained from the FCS experiments. This difference can be attributed to the different experimental and excitation conditions. Particularly, an incomplete recovery of Cy5 to its *all*-*trans* ground state between the excitation laser pulses used in the TRAST-measurements can explain the difference. This in turn depends on the excitation duty cycle, η, and is strongly coupled to a relatively low thermal back-isomerization rate, *k*_*biso*_^*Th*^ (eq 6), in between the excitation pulses in the TRAST experiments (see Supplementary Section S8 for verification). Such incomplete recovery of Cy5 can also be used as a distinguishing parameter, which we used in the flow-based experiments described below.

Next, we performed FCS-measurements of single Cy5- and CF640R-labeled SUVs in different mixtures. Recorded FCS curves, with fitting based on eq 11 and performed as for the free fluorophore mixtures, are presented in Figure 2H. Also for these SUV measurements, a threshold was applied to filter out bursts/spikes in the detected fluorescence intensity time-traces, as described above (and in SI Section S6) before calculating the correlation curves. The generated FCS curves were then fitted to eq 13, with the fitted parameter values from the pure Cy5- and CF640R-labeled SUVs (Figures 2D and 2E) fixed and with the brightness ratio *Q*_*CF*_/*Q*_*Cy*5_ fixed to 1, as previously determined from pure Cy5- and CF640R-labeled SUVs. The fitted *R*_*Cy5*_ values showed an almost linear dependence to the fractions estimated when mixing the pure Cy5- and CF640R-labeled SUV solutions and the concentrations determined for these pure solutions by FCS measurements. Following the same procedure as that for the pure Cy5- and CF640R-labeled SUVs, the corresponding SUV mixtures were then also measured by TRAST (Figure 2I). Fitting the TRAST curves in the same way, applying an exponential model for the fluorescent state populations (eq 14), resulted in a linear dependence on the amplitude with the fraction of Cy5-SUVs, as expected with a brightness ratio of 1 (inset, Figure 2I). Here, the fractions were fixed, based on calculated fractions of mixed solutions of pure Cy5- and CF640R-labeled SUVs, and with the concentrations of these pure Cy5- and CF640R-labeled SUV samples determined from FCS experiments.

### Flow-Based TRAST Measurements

**Figure 3:**
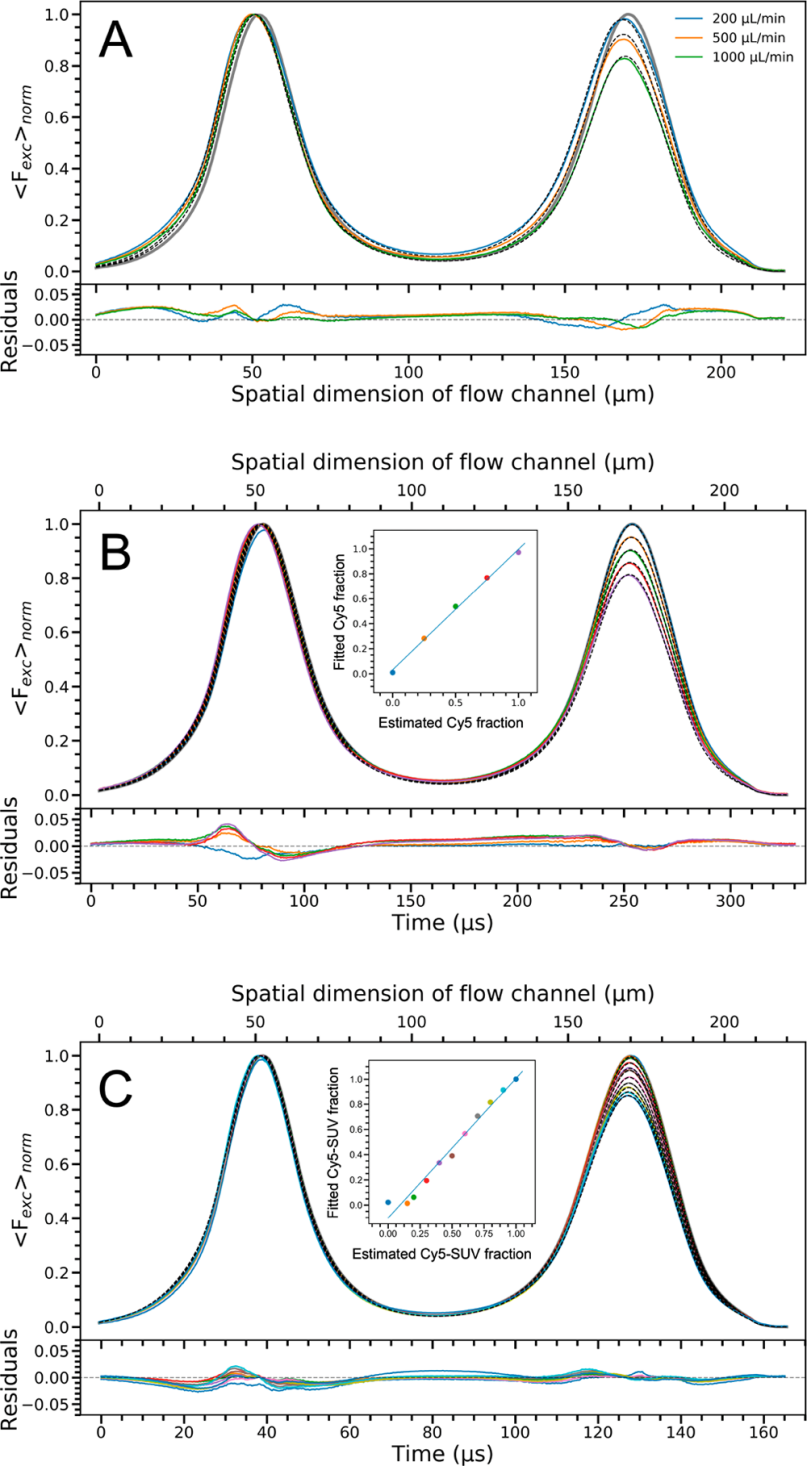
(A) Flow-TRAST curves from free Cy5 (10 nM, PBS), measured at flow rates between 200 and 1000 μL/min (corresponding to 130 to 670 mm/s). Measurements were performed at a peak irradiance of 1.3 kW/cm^2^. At this excitation irradiance and at faster flow rates, larger contrasts between the excitation field profile (gray) and the  curves (colored) were seen in the second excitation profile. This is largely because the thermal relaxation in Cy5, from P back to N, is not fully completed. (B) Flow-TRAST curves of free Cy5/CF640R mixtures in different proportions, measured at a flow rate of 1000 μL/min using the same excitation irradiance as in (A). (C) Flow-TRAST curves of mixtures of Cy5- and CF640R-labeled SUV in different proportions, measured at a flow rate of 2000 μL/min at a peak irradiance value of 3.4 kW cm^2^.

A setup for flow-based TRAST experiments was established and calibrated, as described in the Materials and Methods. Experiments were first performed on free Cy5 in PBS flowing over the two excitation curtain geometries to identify a combination of flow rate, ν, and excitation intensity field, Φ_*exc*_(*x*, *y*), providing a clear relative difference between the recorded Cy5 fluorescence profile  (eq 8) and the laser beam profile, Φ_*exc*_(*t*) (Figure 3A). With higher flow rates a more prominent reduction in  was observed in the second (downstream) excitation curtain. This is attributed to buildup of Cy5 fluorophores in the *cis* state, P, upon passage through the first excitation curtain, and an incomplete recovery to the all-*trans* state N before reaching the second excitation curtain. This is due to the relatively slow thermal back-isomerization rate of Cy5, *k*_*biso*_^*Th*^. Thus, shorter passage times between the excitation curtains (at higher ν) can result in higher populations of P in Cy5 when reaching the second excitation curtain (and a relative drop in the second recorded  curve, green color in Figure 3A).  curves acquired at different flow rates were then fitted to eqs 8 and 9, using the two-state isomerization model of Figure 2C (top). In the fitting of the  curves in Figure 3A, σ_*exc*_, σ_*biso*_, and *k*_*iso*_ were fixed to 6.2 × 10^–16^ cm^2^, 0.15 × 10^–16^ cm^2^, and 29 μs^–1^, respectively, as obtained from FCS measurements (Figure 2A), and only the *k*_*biso*_^*Th*^ rate was globally fitted. The fitted curves were found to well match the experimental curves, yielding a fitted global value of *k*_*biso*_^*Th*^ = 0.0016 μs^–1^, which is a bit lower but in a range similar to that obtained from the stationary TRAST measurements (Figure 2G). The flow-based TRAST measurements on free Cy5 and how  varies with ν are thus in agreement with the Cy5 isomerization kinetics, a slow *k*_*biso*_^*Th*^, and a resulting incomplete recovery of N in-between the excitation curtains. The measurements also indicate that changes in the dark, *cis* state population of Cy5 can be monitored in a microfluidics system, rendering Cy5 (and other cyanine fluorophores with a limited *k*_*biso*_^*Th*^) distinguishing features from other non-isomerizing fluorophores.

To further test this distinguishing feature, flow-TRAST experiments were conducted on free Cy5 dyes mixed with free CF640R dyes in ratios from 0% up to 100% (1000 μL/min (670 mm/s) flow rate, peak irradiance: 1.3 kW/cm^2^). With the low Φ_*exc*_ applied, CF640R fluorophores have a negligible triplet state buildup (given the triplet state parameters determined from FCS, Figure 2B). Thus, no reduction in  is expected in the second excitation curtain (eq 4). For a mixture of Cy5 and CF640R, any reduction in the total  signal in the second excitation curtain (eq 10) can then be attributed to Cy5 and to incomplete thermal back-isomerization of Cy5 in between the excitation curtains (Figure 3B). In the fitting of , the excitation cross sections for Cy5 and CF640R, σ_*CF*_, σ_*Cy*5_, the back-isomerization cross section for Cy5, σ_*biso*_, and *k*_*iso*_ were fixed to 4 × 10^–16^ cm^2^, 6.2 × 10^–16^ cm^2^, 0.15 × 10^–16^ cm^2^, and 29 μs^–1^, respectively. The brightness ratio *Q* = *Q*_*CF*_/*Q*_*Cy*5_ was set to 2.2, as previously found for free dyes from FCS measurements. The *k*_*biso*_^*Th*^ rate was fitted globally for all measurement curves to 0.0012 μs^–1^. This is about five times slower than previously reported for Cy5,^31,32^ as well as compared to the rate estimated from our stationary wide-field TRAST measurements (Supplementary section S8). One possible reason for the slower *k*_*biso*_^*Th*^ rate is that it can be influenced by hydrodynamic interactions from the flowing medium. Depending on activation barriers and conformational properties of the isomerizing compounds and the flow properties, such interactions have been found to generate both enhanced and reduced isomerization rates.^33,34^ Only the fraction of Cy5, *R*_*Cy*5_, was kept free for each measurement sample. The fitted curves, using this limited total number of fitting variables, could well reproduce the experimental  curves, yielding reasonable *R*_*Cy*5_ values and *k*_*biso*_^*Th*^ rate. Similar to the results from the FCS and stationary TRAST measurements, the fit also yielded a linear correlation between the fractions calculated upon preparation of the mixed samples and the fitted fractions, demonstrating that the relative proportions of the two dyes can be experimentally resolved using the flow-TRAST modality.

Next, we studied to what extent single Cy5-labeled SUVs could also be distinguished from CF640R-labeled SUVs by our flow-TRAST procedure. As for the free dye mixture measurements,  curves were acquired for different SUV mixtures, with the fractions of CF640R-labeled SUVs ranging between 0% and 100% (Figure 3C). As expected, for pure CF640R-labeled SUV solutions, no reduction was seen in  within the second excitation curtain, while a gradual relative decrease in this amplitude was observed upon addition of Cy5-labeled SUVs. The recorded  curves were fitted to eq 10, with σ_*CF*_ and σ_*Cy*5_ fixed (4 × 10^–16^ cm^2^ and 6.2 × 10^–16^ cm^2^) and σ_*biso*_ and *k*_*iso*_ fixed to 0.042 × 10^–16^ cm^2^ and 6.2 μs^–1^, as determined from the FCS measurements on the SUVs (Figure 2D). *Q* was set to 1 as found by FCS, while *k*_*biso*_^*Th*^ was globally fitted to 0.0016 μs^–1^. The fraction, *R*_*Cy*5_, of Cy5-labeled SUVs was individually fitted and allowed to vary freely for each SUV mixture. The resulting fitted curves could well reproduce the experimental curves. As for free dye mixture experiments, the fitted *R*_*Cy*5_ values displayed a clear linear dependence to the estimated fractions (inset, Figure 3C). Taken together, flow-TRAST experiments performed on both free dye and SUV mixtures show that spectrally indistinguishable Cy5 and CF640R dyes can be separated in microfluidic measurements based on their different dark transient state properties.

### Wide-Field TRAST Imaging of Bar-Coded HEK293 Cells

**Figure 4:**
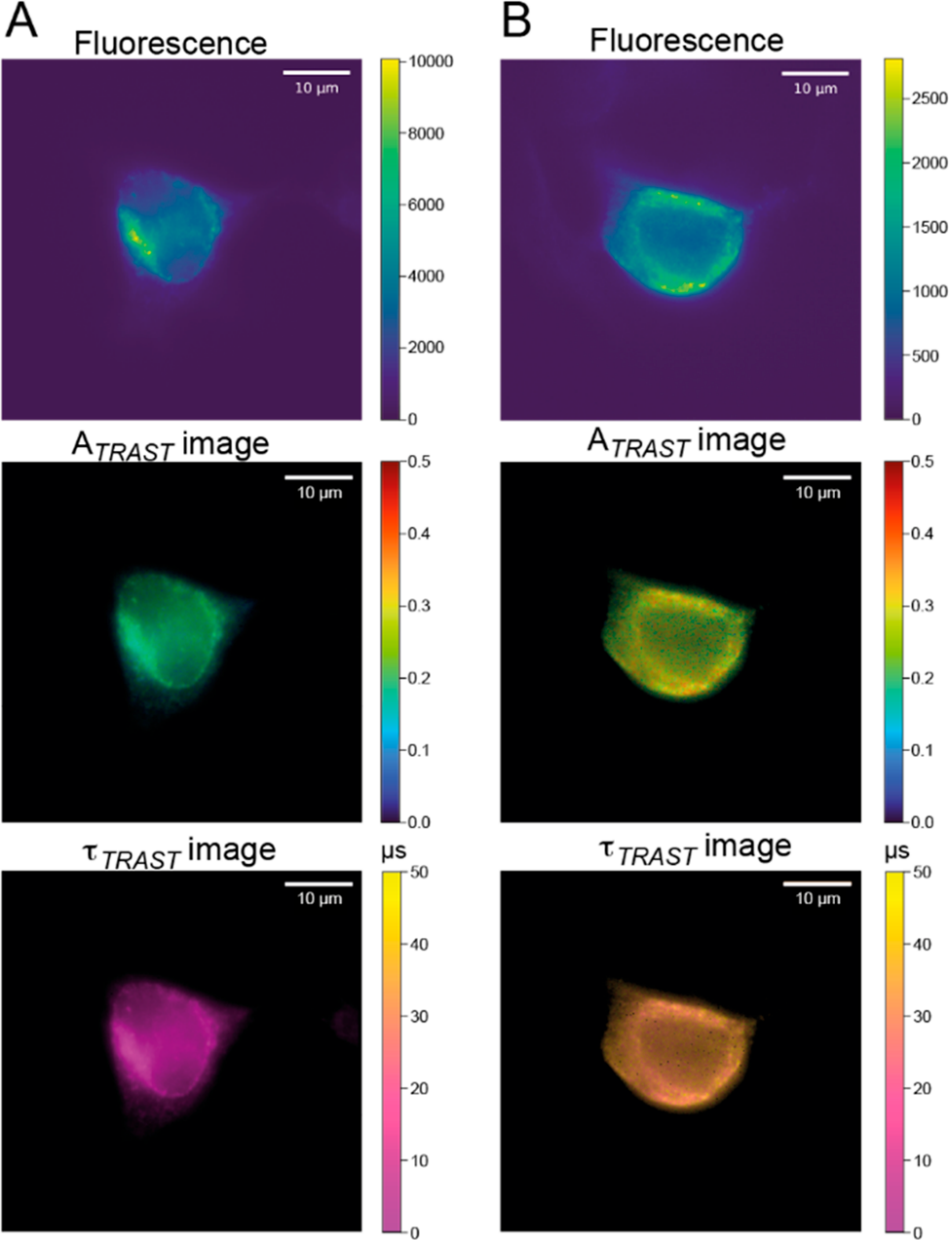
Wide-field TRAST images of HEK293 cells labeled with either AS635(NUP) (A) or Cy5(αT) (B). (A) Middle and bottom: Resulting TRAST images of AS635(NUP) single-labeled cells, showing the spatial dependence of *A*_*TRAST*_ (middle) and of τ_*TRAST*_ (bottom) in the cells. Top: Corresponding fluorescence intensity image. (B) Corresponding images generated from Cy5(αT). As seen from the images in Figures 4A and 4B, there is some difference in how AS635(NUP) (A) and Cy5(αT) (B) distribute within the cell, and there is also a distinct difference in the recorded *A*_*TRAST*_ and in particular the τ_*TRAST*_ values recorded in the AS635(NUP)- and Cy5(αT)-labeled cells (further explored in Figure 5A).

Following the FCS, stationary wide-field, and flow-based TRAST experiments described above, we next investigated to what extent differences in dark state transitions between fluorophores also can be used for bar-coding in cellular imaging. We therefore performed wide-field TRAST imaging on HEK293 cells, with secondary Cy5- and AS635-labeled antibodies targetting primary antibodies against alfatubulin (αT) and nucleoporins (NUPs), respectively (see Materials and Methods). Pixel-wise TRAST curves, with ⟨*F*_*exc*_(*w*)⟩_*norm*_ recorded for 30 different excitation pulse trains with different pulse widths, *w*, were pixel-wise recorded and filtered with a 3 × 3 pixel Gaussian filter (see Materials and Methods). The TRAST curve for each image pixel was then fitted to eqs 1–3, with the emissive singlet state population, [*S*](*t*) in eq 1, given by
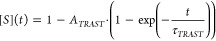
15Here, the overall TRAST relaxation amplitude, *A*_*TRAST*_, and its relaxation time, τ_*TRAST*_, were the only freely fitted parameters. Examples of resulting TRAST images, of both *A*_*TRAST*_ and τ_*TRAST*_, recorded from AS635-labeled NUP and Cy5-labeled αT are shown in Figures 4A and 4B, respectively.

**Figure 5:**
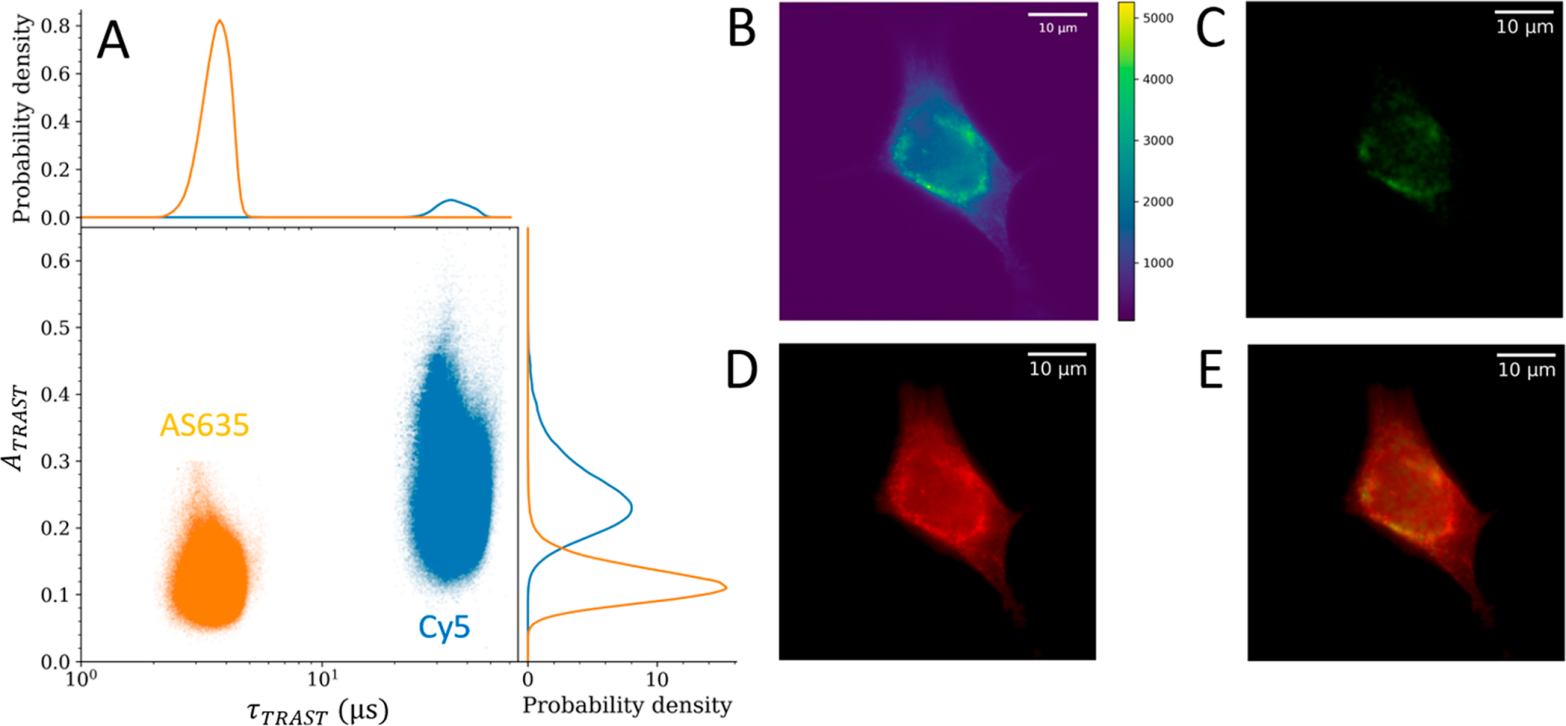
(A) Cumulative 2D histograms of *A*_*TRAST*_ and τ_*TRAST*_ values determined pixel-wise from TRAST images of HEK293 cells, single-labeled with either AS635(NUP) or Cy5(αT) (Figures 4A and 4B). Projections along the *A*_*TRAST*_ and τ_*TRAST*_ axes are the probability density functions (PDFs) for finding a pixel with a certain *A*_*TRAST*_ (*PDF*_*X*_^*A*^(*A*_*TRAST*_)) or τ_*TRAST*_ (*PDF*_*X*_^τ^(τ_*TRAST*_)) value, with X denoting Cy5(αT) (blue lines) or AS635(NUP) (orange lines). The areas under the curves along the τ_*TRAST*_ (logaritmic) and *A*_*TRAST*_ (linear) axes are all normalized to unity. (B) Total fluorescence intensity image of a dual-labeled HEK293 cell. (C) Image of the fluorescence intensity from AS635, extracted from the image in (B) as described in the main text. (D) Corresponding fluorescence intensity image from Cy5. (E) Overlaid image of images in (C) and (D) on the same cell as shown in (B). In images (C) to (E), AS635(NUP) is shown in green, and Cy5(αT) is shown in red.

To account for cell-to-cell variations in *A*_*TRAST*_ and τ_*TRAST*_ and to get a better statistical basis for the fluorophore identification, we next recorded TRAST images from 30 different samples, for each of the Cy5(αT)- and the AS635(NUP)-labeled cells. The resulting, cumulative distributions of the fitted pixel-wise *A*_*TRAST*_ and τ_*TRAST*_ values, from both the Cy5(αT) and the AS635(NUP)-labeled cells, are plotted as two-dimensional (2D) cumulative histograms in Figure 5A. From the 2D histograms, the normalized probability density functions (PDFs) for *A*_*TRAST*_ and τ_*TRAST*_, and for Cy5 and AS635, were then calculated and projected along the axes of *A*_*TRAST*_ and τ_*TRAST*_ (Figure 5A). The 2D histograms of Cy5 and AS635 monolabeled cells can be clearly distinguished from their different (*A*_*TRAST*_,τ_*TRAST*_) distributions, with Cy5(αT)-labeled cells in general yielding higher *A*_*TRAST*_ values (∼0.1–0.6 compared to ∼0–0.3 for AS635(NUP)) and distinctly higher τ_*TRAST*_ values (∼20–50 μs compared to <5 μs for AS635(NUP)).

Next, we used the 2D histogram plots in Figure 5A together with their PDFs as a basis for identification of Cy5(αT) and AS635(NUP) in images from cells labeled with both fluorophores/targets. From the *A*_*TRAST*_ and τ_*TRAST*_ values determined for an individual image pixel from these dual-labeled cells (using eqs 1–3 and 15), we can then determine the fractions of the fluorescence intensity originating from Cy5 and AS635 in that pixel by maximizing a linear combination of their PDFs:
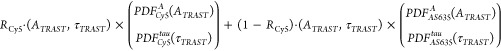
16Here, *PDF*_*j*_^*i*^ represent the projected PDFs along the *A*_*TRAST*_ and τ_*TRAST*_ axes in Figure 5A (with *i* denoting *A*_*TRAST*_ or τ_*TRAST*_) for Cy5 and AS635, respectively (with *j* denoting Cy5 and AS635). In the maximization and fitting of eq 16, *R*_Cy5_ was the only fitted parameter. To generate the separate fluorescence intensities of Cy5 and AS635, the determined fluorescence intensity fractions originating from Cy5 (*R*_Cy5_) and AS635 (1-*R*_Cy5_) in each pixel were then scaled with the total fluorescence intensity recorded in the same pixel. Examples of resulting images of dual-labeled HEK293 cells are shown in Figures 5C-E, displaying the separate fluorescence intensity images of AS635(NUP) (Figure 5C) and Cy5(αT) (Figure 5D), as well as the combined, total fluorescence image of Cy5(αT) and AS635(NUP) (Figure 5E, corresponding to Figure 5B).

The results from wide-field TRAST imaging of the HEK293 cells, and using the presented analysis on both mono-labeled and dual-labeled cell samples, suggest that bar-coding of whole cells is possible exploiting different photophysical kinetic features of spectrally close or even inseparable fluorophores. Moreover, the results also indicate that spatially resolved, multiplexed imaging of the cells for different targets and fluorophores is possible.

## Concluding Remarks

Starting with FCS measurements of the spectrally very similar fluorophores Cy5 and CF640R, free in solution and when labeled to SUVs, we show how these fluorophores differ distinctly in their photoinduced, long-lived, dark state transitions. This suggests that the characteristic photoisomerization feature of Cy5 can be used as an additional, orthogonal fluorescence-based encoding dimension, next to regular fluorescence parameters (intensity, F; emission wavelength, λ_em_; fluorescence lifetime, τ_f_ and anisotropy, FA). It also more generally indicates that different dark state transition features of fluorophores can be used as identifiers, next to microenvironmental sensing applications^16,20,21^ and as a way to enhance signal-to-background conditions.^35−38^ We then applied stationary, wide-field TRAST measurements on the same samples and mixtures and showed that differences in dark state transitions between fluorophores can also be used as a distinguishing feature in measurements not requiring single-molecule detection conditions or high time resolution. Compared to FCS, TRAST measurements are also less sensitive to degree of labeling and aggregation events. This makes TRAST more broadly applicable on different biological samples and thereby also the concept of using dark state transitions as an additional, orthogonal encoding dimension. On this ground, we established a flow-based TRAST concept and showed how the same free fluorophores and SUVs could also be distinguished on-the-fly, in a microfluidic setting. In the current experimental setting, the Cy5 and CF640R samples were mainly distinguished by the dark state recovery of Cy5, via its thermal back-isomerization rate, which was much slower than the triplet decay rate of CF640R. However, using other experimental settings, we expect that differences in other dark state transition rates of fluorophores can also be used as a basis for encoding. To the best of our knowledge, this concept of encoding and the flow-based TRAST concept itself is here described for the first time. This motivates investigations of if transient states can be added as a read-out parameter in flow cytometry or fluorescence activated cell sorting (FACS), a widely used technique to classify cells, vesicles, or exosomes based on their fluorescent properties.^39^ Here, transient state features of fluorophores can be exploited as additional, orthogonal identifying parameters, as shown in this work, but also their environmental sensitivity can be used for flowmetric readouts, reflecting vesicle properties, such as lipid composition and membrane fluidity,^40^ or low-frequency intermittent interactions between fluorophores and dark state quenchers/enhancers in the membranes.^26^ Finally, we also show how this dark state encoding concept can be used with TRAST imaging of cells, further indicating that bar-coding of whole cells using spectrally close or even inseparable fluorophores is possible, as well as spatially resolved, multiplexed imaging of the cells for different targets/fluorophores. The dark state kinetics may be affected by the local environment of the fluorophores. While this can offer a basis for microenvironmental sensing in live cells,^16^ large environmental variations within a sample can also limit the extent to which multiplexing can be applied. Here, the relaxation times originate from two fundamentally different processes; isomerization (Cy5) and triplet state formation (AS635), differing in their relaxation times by more than an order of magnitude and with no overlap in the pixel-wise distributions (Figure 5A). This leaves room for multiplexing also in live cells, with larger variations in the environmental parameters. There is likely also room for multiplexing with additional fluorophores, even if their dark state relaxation parameters would partly overlap, in a similar fashion as e.g. spectral unmixing can be performed on fluorophores with partly overlapping spectra.^41^ Combining linear unmixing of dark state relaxation parameters with spectral or other fluorophore parameters may also represent a strategy to enhance multiplexing. In general, the dark state multiplexing concept presented in this work can be implemented on both stationary and moving samples, onto which well-defined excitation-modulation can applied by laser beam scanning, on–off switching, or translating samples with respect to a laser excitation field.

## Data Availability

All relevant raw data behind this study is available via DOI: 10.5281/zenodo.8006451.

## Supporting Information Available

Section S1: Preparation of fluorophore solutions and lipid vesicles. Section S2: Preparation of HEK293 cells and immunostaining. Section S3: Experimental setup for stationary wide-field TRAST measurements, data acquisition, and analysis. Section S4: Spatial distribution of excitation rates and calculation of the average rates. Section S5: Determination of flow profiling by microfluidic FCS measurements. Section S6: FCS measurements and analysis. Section S7: Fitted rate parameters for Cy5 and CF640 from FCS measurements. Section S8: Recovery of Cy5 from its photoisomerized state P – influence of excitation duty cycle (PDF)
