# Supplementary material for: Fluorescence Bar-Coding and Flowmetry Based on Dark State Transitions in Fluorescence Emitters
Source: J Phys Chem B. 2023 Dec 21;128(1):125–36. doi: 10.1021/acs.jpcb.3c06905 (PMC10788918; doi:10.1021/acs.jpcb.3c06905)
Supplement: Supplementary file 1 — jp3c06905_si_001.pdf [file jp3c06905_si_001.pdf]

Supplementary Information

Fluorescence Bar-coding and Flowmetry based on Dark State Transitions in Fluorescence Emitters

Elin Sandberg[1,+], Baris Demirbay[1,+], Abhilash Kulkarni[1], Haichun Liu[1], Joachim Piguet[1], Jerker Widengren[1,*]

[1]Royal Institute of Technology (KTH), Experimental Biomolecular Physics, Dept. Applied Physics, Albanova University Center 106 91 Stockholm, Sweden

[+] Contributed equally

* Corresponding author: Email: jwideng@kth.se, Phone: +46-8-7907813

Section S1. Preparation of fluorophore solutions and lipid vesicles

Cy5 (mono NHS ester, Cytiva, PA15101) and CF640R (succinimidyl ester, Sigma-Aldrich, SCJ4600044) fluorophores were prepared as 10 µM stock solution in distilled water, and then diluted into a Dulbecco's Buffered Saline (DPBS) buffer (pH = 7.4, Sigma-Aldrich, cat. no. D8537) solution before measurements.For lipid vesicle preparation, POPE (Avanti Polar Lipids, 850757) was labelled with CF640R succinimidyl ester (Sigma-Aldrich, SCJ4600044) and Cy5 Mono NHS Ester (Cytiva, PA15101). Fluorescent dyes were added to POPE with a dye/lipid ratio of 1.5-2 in chloroform (Sigma-Aldrich, 472476) in presence of triethylamine (Sigma-Aldrich, 471283). The solution was stirred for 1-2h at room temperature. Ethanolamine (Sigma-Aldrich, E9508) was added to deactivate unreacted dyes. Each solution was then applied to a TLC Silica Gel 60 plate (Supelco, 1.16835) and developed with chloroform/methanol (Sigma-Aldrich, 32213)/water (65:25:4). The product band was scrapped from the TLC plate and extracted with a 3:1 choloroform/methanol solution. The dye-labeled lipids were then used to prepare small unilamellar vesicles (SUVs), as previously described [1]. In short, POPC in chloroform (1.74 µmol, Avanti Polar Lipids, Inc.) was dried under a gentle flow of N_2 for about 20 min. After complete removal of any residual solvent, 1.4 ml DPBS buffer was added to the dried dye lipids, vortexed for 1 min, and then sonicated at 0 ^{o}C for 10 min by a Branson SFX250 sonicator at 50% duty cycle (0.50 s on/off), 50% power (125 W) and a 1/8" microtip (Emerson Electric Co, St. Louis, MO, USA). After sonication, the solution was centrifuged for 20 min at 14000 g, and the supernatant was then filtered by using a 0.2 µm spin-filter (Corning, NY, USA) to remove large aggregates. The fraction of labelled POPE lipids in the SUVs were typically lower than 1/50000, to prevent having more than one fluorophore per SUV.

Section S2. Preparation of HEK293 cells and immunostaining

HEK293 kidney cells (ATCC) were maintained in DMEM/F-12 medium (Gibco) supplemented with 10% fetal bovine serum (VWR, Biowest), 1% penicillin-streptomycin (Gibco). The cells were first cultured in a 25 cm^3flask and passaged every 3 days with Trypsin-EDTA (Biowest). ~ 15×10^3 cells were grown in glass-bottom 8-well cell culture plates (Nunc Lab-Tek II Chambered Coverglass) 24 h before stationary TRAST measurements.

For the immunostaining, the cell culture medium was first removed and HEK293 cells were washed 3 times with DPBS. The cells were fixed and permeabilized with 3.7% paraformaldehyde (PFA), 0.1% glutaraldehyde and 0.5% Triton X for 20 min, and then washed 3 times with DPBS. Afterwards, the cells were blocked for nonspecific binding with 2% w/v bovine serum albumin (Sigma, Sweden) for about 40 min and washed three times with DPBS. The cells were incubated with primary antibodies (1:200) for 1 h. Rabbit polyclonal antibodies (Invitrogen) were used for targeting nucleoporin Nup153 while Mouse monoclonal antibodies (Invitrogen) were used against alpha tubulin of HEK293 cells. The cells were washed three times with DPBS before incubating with labelled secondary antibodies for an hour (goat anti-rabbit and goat anti-mouse (Sigma), conjugated with Abberior Star 635 and Cy5 fluorophores, respectively). The labelled cells were washed 3 times and DPBS was used as an observation medium for experiments.

Section S3. Experimental setup for stationary wide-field TRAST measurements, data acquisition and analysis.

TRAST measurements were carried out on a home-built TRAST setup, as previously described [1, 2], based on an inverted epi-fluorescence microscope (Olympus, IX70). Fluorescence was excited by a 638 nm diode laser (Cobolt, 06-MLD, 240 mW) using an excitation filter (Semrock BrightLine 637/7). The laser beam was modulated by an acousto-optic modulator (AOM; AA Opto Electronics, MQ180-A0,25-VIS), reflected by a dichroic mirror (ZT640rdc) and then focused close to the back aperture of the objective (Olympus, UPLSAPO 60x/1.20 W) to produce a wide-field illumination in the sample (beam waist ω_0 = 10-25 µm ($1/e^2$ radius). The fluorescence signal was collected by the same objective, passed through the same dichroic mirror and an emission filter (ET706/95m, Chroma) before detection by a sCMOS camera (Hamamatsu ORCA-Flash4.0 V3). The experiments were controlled and synchronized by custom software implemented in Matlab. A digital I/O card (PCI-6602, National Instruments) was used to trigger the camera and generate random excitation pulse trains sent to the AOM driver unit.

In the data acquisition, a complete TRAST experiment consisted of a stack of 30 fluorescence images. Each image represents the total fluorescence signal from an entire excitation pulse train, captured using a camera exposure time of $t_{exp} = t_{ill}/\eta$. Pulse durations, w, were distributed logarithmically between 100 ns and 10 ms and were measured in a randomized order to avoid bias due to time effects. An additional 10 reference frames, all using 100 ns pulse duration (w_{short}) to avoid dark state build-up, were inserted at regular intervals between the 30 main images to track any permanent bleaching of the sample.

The TRAST data was analyzed by software implemented in Matlab, as previously described [2, 3]. Recorded TRAST data was first pre-processed by subtraction of static ambient background, by optional binning to either larger pixels or regions of interest (ROIs) within the recorded images, and by correction for bleaching. The bleaching correction was based on 10 reference frames, recorded in between the regular frames throughout the measurements. The overall bleaching was maximally 5-10 % of the total detected intensity in the experiments.

In all measurements, TRAST curves were produced by calculating $\langle F_{exc}(w) \rangle_{norm}$ within a region of interest (ROI) corresponding to a 15 µm radius in the sample plane, centered on the excitation beam, for both the vesicle and live cell measurements. Within the selected ROI, an

average excitation rate was then calculated, as described in section S4. Fitting of photophysical parameters was then performed by simulating theoretical TRAST curves using Eqs. (1-3) and comparing them to the experimental data. The set of parameters best describing the experimental data was then found using non-linear least squares optimization.

Section S4. Spatial distribution of excitation rates & calculation of the average rates

The non-uniform shape of the excitation beam means that the excitation photon flux, $\Phi_{exc}(\bar{r})$, is a function of position in the sample. As a consequence, a detailed TRAST analysis should include a spatial dependence to both the excitation rates and the resulting electronic state populations. The total fluorescence signal on each pixel of the camera then becomes a convolution of $S_1(t)$ and the microscope collection efficiency function, $CEF(\bar{r})$, as shown in Eq. (1). However, simulating the whole 3D sample volume, and computing the projected 2D image on the camera, becomes a costly operation when performed in each iteration of the fitting algorithm. While this procedure is possible, and sometimes required, pre-computing an average observed excitation rate, $\hat{k}_{01}$, for each pixel or ROI to be analyzed, speeds up the fitting significantly, without appreciable loss of accuracy. The approximate $\hat{k}_{01}$ is computed once, before fitting starts, by weighting $k_{01}(\bar{r})$ by brightness and collection efficiency, , in the following manner

$$\hat{k}_{01} = \frac{\iiint k_{01}(\bar{r}) \cdot \hat{S}_1(\bar{r}) \cdot CEF(\bar{r})\, dV}{\iiint \hat{S}_1(\bar{r}) \cdot CEF(\bar{r})\, dV} \qquad (S1)$$

Above equation, $\hat{S}_1(\bar{r}) = k_{01}(\bar{r}) / \left(k_{01}(\bar{r}) + k_{10}\right)$ represents the population of S at onset of excitation, after equilibration between the singlet states S_0 and S_1, but before build-up of the other states.

Section S5. Determination of flow profiling by microfluidic FCS measurements

To determine the flow profiling in flow-based TRAST measurements, complementary FCS experiments were performed with a microfluidic system. By scanning the position of the excitation laser with 5 µm spatial resolution across the flow direction in the middle height (that is 25 µm) of the flow channel, 98 different FCS curves were recorded for 10 nM of CF640R dye in PBS under a constant flow rate (40 µL/min) by using a mechanical syringe pump. In microfluidic FCS measurements, excitation power is kept low at 84 µW to avoid the triplet state build-up. Some example FCS data is visualized in Figure S2A where all experimental FCS curves were fitted to a modified correlation function as given below [4]:

$$G(\tau) = \frac{1}{N} G_D(\tau) \cdot \exp\left[-\left(\frac{\tau}{\tau_{flow}}\right)^2 \cdot G_D(\tau)\right] \qquad (S5)$$

$$G_D(\tau) = \left(1 + \frac{\tau}{\tau_D}\right)^{-1} \left(1 + \left(\frac{w_0}{z_0}\right)^2 \frac{\tau}{\tau_D}\right)^{-\frac{1}{2}} \qquad (S6)$$

where τ_{flow} is the flow time of the CF640R dyes in microfluidic channel. In curve fitting, N and τ_{flow} were fitted as free parameters whereas τ_D is fixed to 50 µs (as determined from the FCS curve recorded at 40 µL/min flow rate). With the knowledge of w_0 and τ_{flow} values, the flow speed V_{flow} at different coordinates is calculated using $V_{flow} = w_0/\tau_{flow}$. The fitted N and τ_{flow} values together with calculated V_{flow} were plotted in Figure S2(B-D). For FCS curves recorded at the coordinates of -240 µm and 240 µm, smaller FCS amplitudes were found most likely due to the sticking of dye molecules to the interior walls of microfluidic chip (the flow channel width is 500 µm). Therefore, N values were found to be higher for the coordinates closer to the channel walls (see Figure S2B). However, in the middle region of the flow channel between -180 µm and 180 µm, the fitted τ_{flow} and calculated V_{flow} values were found to be fairly flat, demonstrating a uniform laminar flow profile. In flow-based TRAST experiments, as the excitation beam curtains are well positioned in between these coordinates, the flow profile is treated as laminar flow.

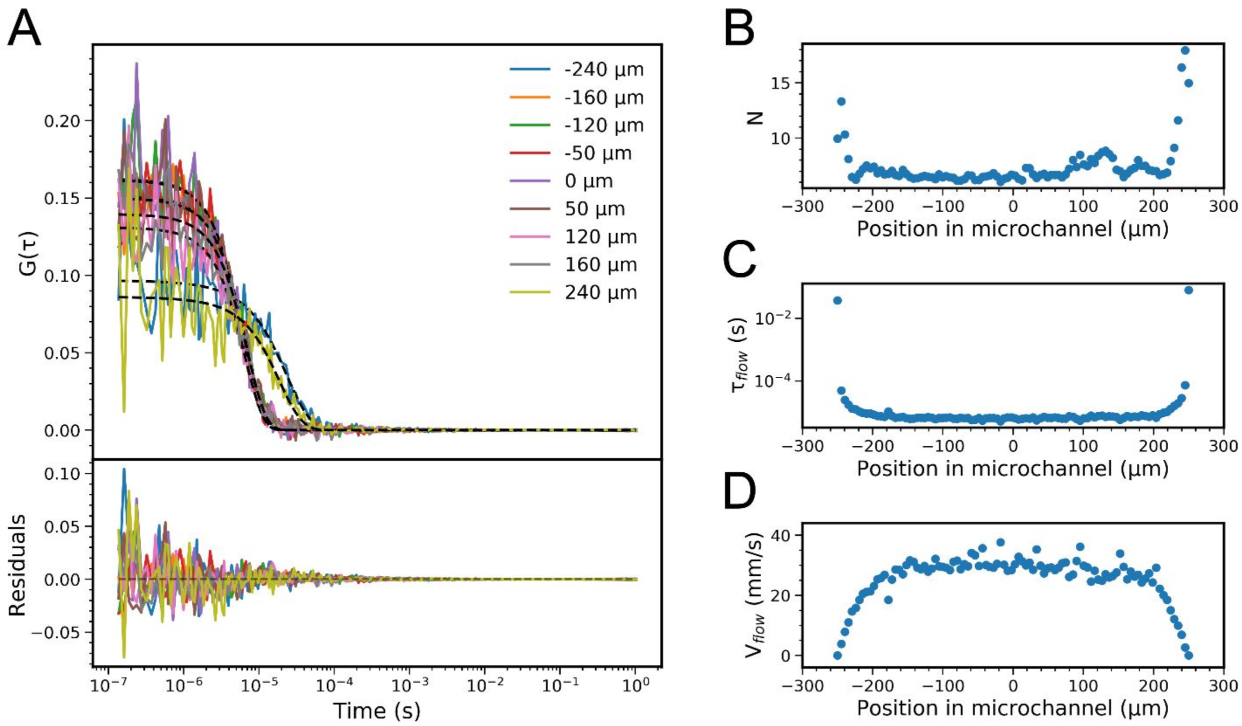

Figure S1. FCS measurements performed with microfluidic flow system. (A) Experimental FCS curves recorded at different coordinates across the flow direction. (B) Number of molecules in the detection volume, (C) flow time, τ_{flow} and (D) flow speed, V_{flow} of CF640 molecules in microfluidic channel as obtained from the fitting of FCS curves at different channel coordinates.

Section S6. FCS measurements and analysis

FCS measurements were performed on a commercial, epi-illuminated, confocal laser scanning microscope (Olympus FV1200), with the samples excited by the focused beam (focal radius of 338nm $1/e^2$ radius) of a 640 nm diode laser (LDH-D-C-640, PicoQuant GmbH, Berlin) in continuous wave. The emitted fluorescence was collected back through the microscope objective (UPlanSApo 60x/1.2w, Olympus), passed a dichroic mirror, (ZT405/488/635rpc-UF2, Chroma) an emission filter (HQ720/150, Chroma for emission), and was focused onto a pinhole (50 μm) in the back focal plane. The fluorescence signal was finally split and directed on two avalanche photodiodes (Tau-SPAD, PicoQuant GmbH, Berlin), whose signals were collected by a data acquisition card (Hydraharp 400, Picoquant, Berlin). In the FCS measurements, for freely diffusing fluorescent molecules undergoing dark state transitions, the autocorrelation curves of the recorded fluorescence intensity, $F(t)$, can be described by:

$$G(\tau) = \frac{< F(t)F(t+\tau) >}{< F(t) >^2} = G_D(\tau)G_T(\tau) + 1 \tag{S2}$$

where $G_D(\tau)$ denotes the translational diffusion-dependent part $G_T(\tau)$ signifies the contribution from photo-induced dark state transitions. $G_D(\tau)$ can be expressed as:

$$G_D(\tau) = \frac{1}{N_m}\left[1 + \frac{\tau}{\tau_D}\right]^{-1} \times \left[1 + \left(\frac{\omega_0}{\omega_z}\right)^2 \frac{\tau}{\tau_D}\right]^{-\frac{1}{2}} \tag{S3}$$

with $\boldsymbol{\omega_0}$ and $\boldsymbol{\omega_Z}$ denoting the distances from the center of the laser beam focus in the radial and axial direction respectively at which the collected fluorescence intensity has dropped by a factor of $\boldsymbol{1/e^2}$ compared to its peak value. $\boldsymbol{N_m}$ is the mean number of fluorescent molecules within the detection volume. $\boldsymbol{\tau_D}$ is the characteristic diffusion time of the fluorescent molecules, given by the diffusion coefficient $\boldsymbol{D}$ as $\boldsymbol{\tau_D = \omega_0^2/4D}$.

If no dark state transitions occur, the blinking term $G_T(\tau) = 1$. Otherwise, for a fluorophore with n dark transient states, and for τ much longer than the anti-bunching relaxation times of the fluorophores, $G_T(\tau)$ can be expressed as a normalized set of relaxation terms [5, 6], averaged over the confocal detection volume, weighted by the square of the detected molecular brightness of the molecules, $W(\bar{r})$:

$$G_T(\tau) = \frac{\int W^2(\bar{r})\left[1 - \sum_{i=1}^{n}\left[A_i(\bar{r}) - A_i(\bar{r})e^{-\lambda_i(\bar{r})\tau}\right]\right]dV}{\int W^2(\bar{r})\left[\sum_{i=1}^{n}[1 - A_i(\bar{r})]\right]dV}$$

(S4)

Here, $\lambda_i(\bar{r})$ are the eigenvalues and $A_i(\bar{r})$ the related amplitudes, reflecting the population build-up of the different photo-induced non-fluorescent states. At steady state and with no photobleaching, the sum of the population probabilities for S_0 and S_1, together with $\sum_{i=1}^{n}[A_i(\bar{r})]$ equals one.

For FCS-analysis of single Cy5- and CF640R-labelled SUVs alone and in different mixtures, the Sympohotime software (Picoquant, Berlin) and an upper threshold was applied to filter out bursts/spikes in the detected fluorescence intensity time-traces before calculating the FCS curves. The threshold was set to 7 standard deviations above the mean fluorescence intensity. Aggregates or multi-labelled SUVs passing through the FCS detection volume, can result in lower dark state relaxation amplitudes than from the single-labelled, not aggregated SUVs. Since they would also contribute with their brightness squared to the experimental FCS curves (Eq. 13), the use of filtered FCS curves allowed this contribution to be minimized and made the estimation of the relative concentrations of Cy5 and CF640R more reliable.

For FCS-analysis of single Cy5- and CF640R-labelled SUVs alone and in different mixtures, the Sympohotime software (Picoquant, Berlin) and an upper threshold was applied to filter out bursts/spikes in the detected fluorescence intensity time-traces before calculating the FCS curves. The threshold was set to 7 standard deviations above the mean fluorescence intensity. Aggregates or multi-labelled SUVs passing through the FCS detection volume, can result in lower dark state relaxation amplitudes than from the single-labelled, not aggregated SUVs. Since they would also contribute with their brightness squared to the experimental FCS curves (Eq. 13), the use of filtered FCS curves allowed this contribution to be minimized and made the estimation of the relative concentrations of Cy5 and CF640R more reliable.

Section S7. Fitted rate parameters for Cy5 and CF640 from FCS measurements.

Cy5 (free):

$\boldsymbol{k_{isc}}$: 1.1μs^{-1} fixed (from ref 5)

$\boldsymbol{k_T}$: 0.5μs^{-1} fixed (from ref 5)

$\boldsymbol{k_{iso}}$: 29μs^{-1}

$\boldsymbol{\sigma_{biso}}$: 0.15μs^{-1}

Cy5 (SUVs):

$\boldsymbol{k_{isc}}$: 1.1μs^{-1} fixed (from ref 5)

$\boldsymbol{k_T}$: 0.5μs^{-1} fixed (from ref 5)

$\boldsymbol{k_{iso}}$: 6.2μs^{-1}

$\boldsymbol{\sigma_{biso}}$: 0.15 μs^{-1}

CF640R (free and SUVs):

$\boldsymbol{k_{isc}}$: 0.7μs^{-1}

$\boldsymbol{k_T}$: 0.5μs^{-1}

Section S8. Recovery of Cy5 from its photo-isomerized state P – influence of excitation duty cycle.

While recording TRAST-curves of Cy5, it was noted that the amplitudes of the curves were generally reduced compared to the dark state relaxation amplitudes obtained in the FCS measurements (Figure 2F versus Figure 2H). One possible reason for this is incomplete dark state relaxation in-between the excitation pulses applied in the TRAST measurements. In the stationary wide-field TRAST experiments, the duty-cycle, η, was set to 0.01. For most fluorophores, this gives sufficient time for their dark transient states to fully recover back to the emissive singlet state. At our experimental conditions, it was found that for Cy5, the back-isomerization rate is predominantly excitation-driven (Eq. 10), with only a lower thermal recovery rate, k_{biso}^{Th}, effective in-between the pulses. To investigate to what extent η affects the dark state relaxation amplitudes in the recorded TRAST curves and may lead to incomplete recovery from P to N in-between the excitation pulses, TRAST-curves were recorded with different η. At our experimental conditions, it was found that η at around 0.001 was required for the TRAST-curves to converge to similar data points (Figure S1A). An incomplete recovery back to the ground state through thermal relaxation can likely explain the reduction in amplitude observed for higher η. Figure S1B shows how the normalized average fluorescence varies with η for different pulse-widths, *w*, confirming the convergence of data-points for η <0.001 in Figure S1A. One can also note that η primarily influences the shorter *w* data points, with the time in-between pulses then shorter and giving less time for ground state recovery. In Figure S1C, the S_1 population of Cy5 is calculated based on the fitted isomerization rates, as obtained by FCS, with a thermal rate of $k_{th} = 0.005$ μs^{-1} and with the same duty-cycles as was used for the measurements in Figure S3A. The calculated curves in Figure S1C show a similar behavior as the experimental curves (Figure S1A), which indicates that an incomplete recovery due to a low k_{biso}^{Th} is a likely reason for the decreased amplitudes observed. Using the same experimental parameter values for the calculations, Figure 3D shows how the population of the ground state of N, S_{0N}, at onset of the next excitation pulse in a pulse train, depends on η.

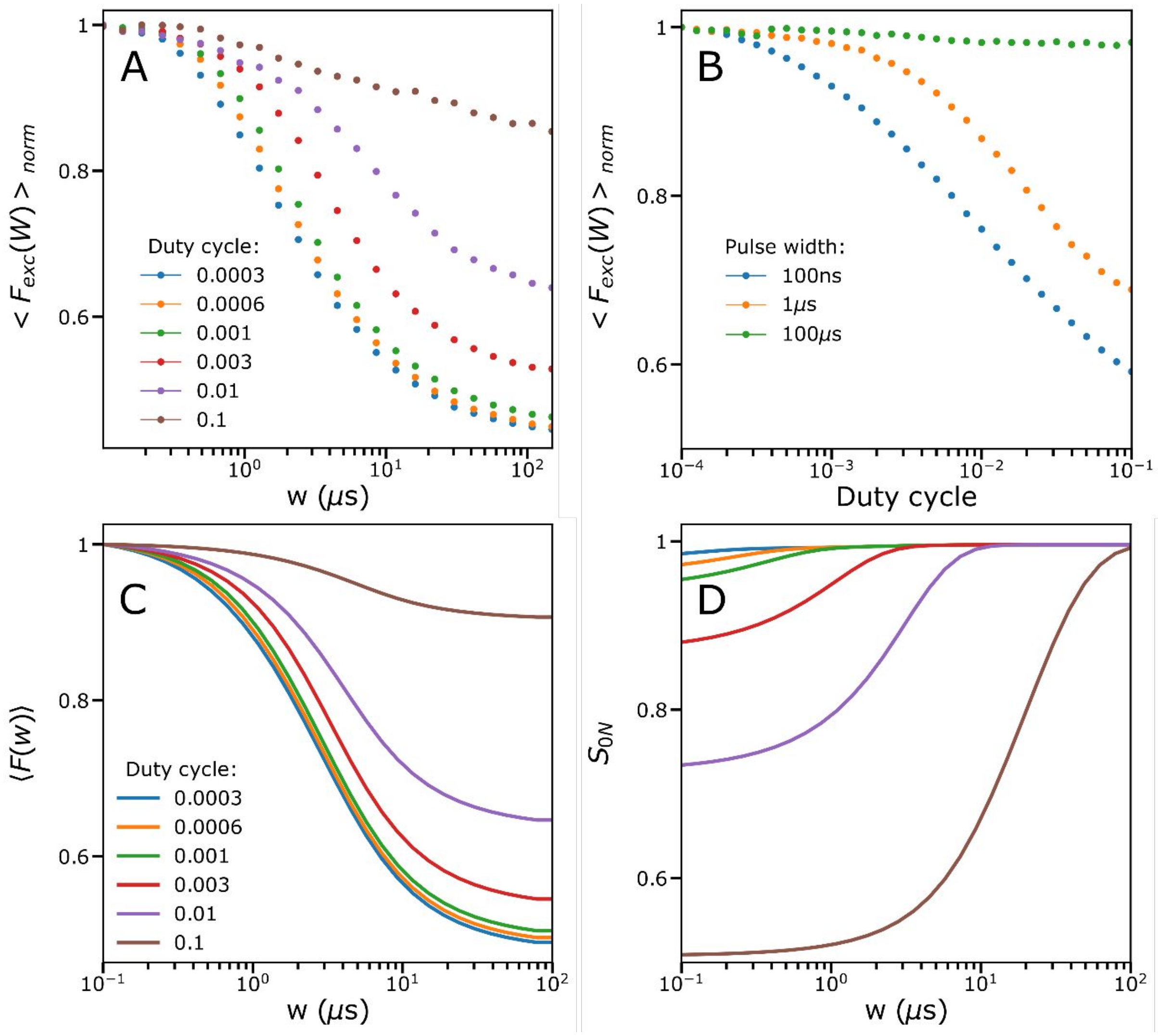

Figure S2. (A) TRAST curves recorded from Cy5 in solution with different η applied (indicated in the legend). Here, $\Phi_{exc} = 4.6$ kW/cm². (B) TRAST measurements of Cy5 in solution with different fixed pulse-widths (indicated in legend), versus η ($\Phi_{exc} = 4.6$ kW/cm²). (C) Calculated TRAST-curves for different η, based on fitted isomerization rate parameter values determined from FCS, and with $k_{biso}^{Th} = 0.005$ μs^{-1}. (D) Initial populations of S_{0N} for different η versus *w*.

References

1. Du, Z.X., et al., *Imaging Fluorescence Blinking of a Mitochondrial Localization Probe: Cellular Localization Probes Turned into Multifunctional Sensors br.* Journal of Physical Chemistry B, 2022. **126**(16): p. 3048-3058.
2. Tornmalm, J., et al., *Imaging of intermittent lipid-receptor interactions reflects changes in live cell membranes upon agonist-receptor binding.* Scientific Reports, 2019. **9**.
3. Tornmalm, J. and J. Widengren, *Label-free monitoring of ambient oxygenation and redox conditions using the photodynamics of flavin compounds and transient state (TRAST) spectroscopy.* Methods, 2018. **140**: p. 178-187.
4. Gösch, M., et al., *Hydrodynamic flow profiling in microchannel structures by single molecule fluorescence correlation spectroscopy.* Analytical Chemistry, 2000. **72**(14): p. 3260-3265.
5. Widengren, J., U. Mets, and R. Rigler, *FLUORESCENCE CORRELATION SPECTROSCOPY OF TRIPLET-STATES IN SOLUTION - A THEORETICAL AND EXPERIMENTAL-STUDY.* Journal of Physical Chemistry, 1995. **99**(36): p. 13368-13379.
6. Widengren, J. and P. Schwille, *Characterization of photoinduced isomerization and back-isomerization of the cyanine dye Cy5 by fluorescence correlation spectroscopy.* Journal of Physical Chemistry A, 2000. **104**(27): p. 6416-6428.
